# Course of the Metabolic Syndrome (Mets) in a First Episode Psychosis Sample

**DOI:** 10.1192/j.eurpsy.2022.639

**Published:** 2022-09-01

**Authors:** E. Garcia De Jalon, L. Aranguren, A. Fernandez Falces, A. Aquerreta Unzue, N. Pereda

**Affiliations:** SERVICIO NAVARRO DE SALUD, Programa De Primeros Episodios, PAMPLONA, Spain

**Keywords:** First-episode psychosis, metabolic syndrome, antipsychotic treament

## Abstract

**Introduction:**

There is evidence that metabolic syndrome (MetS) is common in chronic psychosis but also exists in the early stages.

**Objectives:**

To study the prevalence and course of MetS over a period of 2 year after a first episode psychosis. To determine whether there may be differences in its prevalence according to the type of antipsychotic used over two years.

**Methods:**

A sample of 300 patients participate in the PEPsNa Early Intervention Programme. SMet was determined at baseline and at 6, 12, 18 and 24 months. The type of antipsychotic used at each assessment moment is collected (none, aripiprazole, paliperidone, others). Adult Treatment Panel III (ATP III) criteria were used to define MetS.

**Results:**

The prevalence of MetS at baseline is 4.44% and increases to 7.96% at 6 months, 10.1% at 12 months, 8.62% at 18 months and 9.01% at 24 months. The prevalence of MetS increases at 6 (p<0.021) and 12 months (p<0.003) compared to baseline and then remains stable. Only at 6 months assessment there are significant differences (F-Ficher p<0.022) in the presence of MetS (15.8%) in the paliperidone group treatment (oral or LAI).

**Conclusions:**

Metabolic syndrome (MetS) exists from the early stages of psychosis and increases in the first 6-12 months and remains stable thereafter. The type of antipsychotic treatment only seems to have an influence at 6 months, with no differences at other follow-up times.

**Disclosure:**

No significant relationships.

